# Reference evapotranspiration of Brazil modeled with machine learning techniques and remote sensing

**DOI:** 10.1371/journal.pone.0245834

**Published:** 2021-02-09

**Authors:** Santos Henrique Brant Dias, Roberto Filgueiras, Elpídio Inácio Fernandes Filho, Gemima Santos Arcanjo, Gustavo Henrique da Silva, Everardo Chartuni Mantovani, Fernando França da Cunha

**Affiliations:** 1 Agronomy Department, Ponta Grossa State University (UEPG), Ponta Grossa, Paraná, Brazil; 2 Agricultural Engineering Department, Federal University of Viçosa (UFV), Viçosa, Minas Gerais, Brazil; 3 Soil Department, Federal University of Viçosa (UFV), Viçosa, Minas Gerais, Brazil; 4 Polytechnic School, Federal University of Bahia (UFBA), Salvador, Bahia, Brazil; Universiti Teknologi Malaysia, MALAYSIA

## Abstract

Reference evapotranspiration (ETo) is a fundamental parameter for hydrological studies and irrigation management. The Penman-Monteith method is the standard to estimate ETo and requires several meteorological elements. In developing countries, the number of weather stations is insufficient. Thus, free products of remote sensing with evapotranspiration information must be used for this purpose. In this context, the objective of this study was to estimate monthly ETo from potential evapotranspiration (PET) made available by MOD16 product. In this study, the monthly ETo estimated by Penman-Monteith method was considered as the standard. For this, data from 265 weather station of the National Institute of Meteorology (INMET), spread all over the Brazilian territory, were acquired for the period from 2000 to 2014 (15 years). For these months, monthly PET values from MOD16 product for all Brazil were also downloaded. By using machine learning algorithms and information from WorldClim as covariates, ETo was estimated through images from the MOD16 product. To perform the modeling of ETo, eight regression algorithms were tested: multiple linear regression; random forest; cubist; partial least squares; principal components regression; adaptive forward-backward greedy; generalized boosted regression and generalized linear model by likelihood-based boosting. Data from 2000 to 2012 (13 years) were used for training and data of 2013 and 2014 (2 years) were used to test the models. The PET made available by the MOD16 product showed higher values than those of ETo for different periods and climatic regions of Brazil. However, the MOD16 product showed good correlation with ETo, indicating that it can be used in ETo estimation. All models of machine learning were effective in improving the performance of the metrics evaluated. Cubist was the model that presented the best metrics for r^2^ (0.91), NSE (0.90) and nRMSE (8.54%) and should be preferred for ETo prediction. MOD16 product is recommended to be used to predict monthly ETo, which opens possibilities for its use in several other studies.

## Introduction

Adequate water availability is essential to ensure the sustainability of the environment and the various human activities. Therefore, their sustainable management is necessary, as water is a finite resource in quality and quantity [1].

The irrigated agriculture accounts for about 70% of all freshwater used by humans [2, 3]. Thus, it is necessary to study the water demand of crops [4]. The most relevant component of the terrestrial phase of the hydrological cycle is evapotranspiration (ET) [5], which is crucial for the management of water resources [6–8].

To calculate the reference evapotranspiration (ETo), in the management of water use, the Food and Agriculture Organization (FAO) recommends using the Penman-Monteith method (PM-FAO) [9]. However, PM-FAO requires a large number of meteorological variables, such as: solar radiation, air temperature, wind speed, and relative humidity [10, 11]. These variables are often difficult to obtain, as the necessary sensors are very expensive, making it even more difficult to calculate ET, especially when the goal is to obtain its spatial dynamics [11–13].

In Brazil, the National Institute of Meteorology (INMET) provides free meteorological data from a large network of stations distributed throughout the country. The historical data of 265 conventional weather stations can be accessed through the Meteorological Database for Teaching and Research (BDMEP). Considering that each meteorological station represents an area with a radius of 50 km [9], meteorological information for a maximum area of 2,081,300 km^2^ would be possible. It is worth mentioning that this area is a potential area, as overlapping between areas of two or more meteorological stations and different conditions of the microclimate was disregarded. Even so, INMET stations correspond to less than 25% of the area in Brazil, which is 8,514,817 km^2^. Given the above, it is clear that existing weather stations in Brazil do not have the capacity to represent the behavior of meteorological data across the country, which requires the search for alternative techniques that aim to overcome these problems.

One alternative is to use spatial products from satellite images, since the measurements of surface variables with these products can be dense in time and space. With this technique, it is possible to monitor large areas quickly and at a moderate cost. The use of remote sensing in agriculture and hydrology has gained momentum in recent years, mainly due to the development of new orbital sensors and the availability of free images that can be used in hydrological and climatic monitoring [14–20].

Several authors have attempted to develop ET products for application in water resource management [21–23]. The ability to use information from satellite sensors to estimate ET has been developing rapidly and offers the opportunity to understand how ET behaves in space and time, thus reducing the uncertainty levels of this parameter [12, 13, 24].

One of the available ET products is MOD16 [25, 26], which provides four parameters, being the potential evapotranspiration (PET) one of them. This product is obtained indirectly from other products of the MODIS (Moderate Resolution Imaging Spectroradiometer) sensor, along with meteorological information [27] from data taken from a global meteorological network.

Some authors such as Kim et al. [28], Polhamus et al. [29] and Westerhoff [30], who worked with the MOD16 product, found that the original product data had values that overestimate those measured at meteorological stations, which had already been confirmed by Mu et al. [26], authors of the product. Kim et al. [28] found underestimations in PET at a cropland site; however, the MOD16 product successfully depicted the general pattern of the PET. Westerhoff [30] found slight overestimates when compared with the values obtained by the standard PM-FAO method in cold months, and this discrepancy increased in the warmer months. So, we have the suspicion that the MOD16 PET obtained in the Brazilian territory also presents higher values in relation to the ETo of PM-FAO.

Thus, in addition to good covariates, it is important to use models capable of predicting ETo with high performance. Among these models, the following are worth mentioning: multiple linear regression (LM), cubist, random forest (RF), partial least squares (PLS); principal components regression (PCR); adaptive forward-backward greedy (FoBa); generalized boosted regression (GBM), and generalized linear model by likelihood-based boosting (GLMboost). More information about these methods can be obtained in the item “Regression algorithms and modeling” in the material and methods item of this article.

Studies using PET (MOD16) data and machine learning models to estimate ETo for the entire Brazilian territory have not yet been carried out. Spatialized ETo information will contribute to the water management of water crops and to the irrigation project. Due to the low density of meteorological stations in the Brazilian territory, ETo values from very distant locations are used to calculate the project irrigation depth. Thus, one of the applications of our research would be to provide ETo information for every 1 km^2^ (MOD16 spatial resolution) for the entire Brazilian territory. This would contribute to better designed irrigation systems, giving greater confidence in the equipment and lower costs.

Considering the need for rational use of water, from an economic and environmental point of view, also taking into account the need to estimate ETo values accurately in time and space, we believe that the MOD16 product provides an alternative to solve this problem. The objective of the present study was to model and make available, using machine learning algorithms, the spatial distribution of ETo for the Brazilian territory, using as covariates the WorldClim dataset and the PET (MOD16).

## Material and methods

### Characterization of the study area

The study was conducted for the whole territory of Brazil, the fifth largest country in the world, with an area of 8.5 million km^2^. The geographic location of the country causes it to receive a high incidence of solar radiation on the surface; therefore, the predominant climate is tropical. However, there are other climatic groups, such as temperate and dry [31].

The climatic classification throughout the Brazilian territory, according to the classification of Alvares et al. [31] is presented in S1 File. The classifications that begin with “A” deal with a type of tropical climate with some characteristics similar to those of megathermal climates: average temperature of the coldest month of the year greater than 18°C, absent winter season, and strong annual precipitation (superior to the maximum potential ET of the 12 months). The subdivision of the tropical climate A is made from the precipitation, being: Af—Equatorial, Am—Monsoon, As—Savanna, Winter rain, and Aw—Savanna, Summer rain [31].

Classifications that start with B are related to the type of arid climate and have characteristics such as: Dry climates (annual rainfall less than 500 mm), maximum annual potential ET superior to the annual precipitation, and there are no permanent water courses. The subdivision of the arid climate (B) is also made from the precipitation: BS–Steppe climate, but there is only one classification of B in Brazil, BSh—Arid Climate, Steppe with annual precipitation between 380 mm and 760 mm, dry and hot, with the following characteristics: Average annual air temperature greater than 18°C and desert or hot semi-desert (average annual air temperature of 18°C or greater).

Classifications that start with C refer to the type of temperate or hot temperate climate, with characteristics such as: Mesothermal climates, average air temperature of the coldest three months between -3°C and 18°C, average temperature of the warmest month higher than 10°C, and well-defined summer and winter seasons. The subdivision (second letter) of the hot temperate climate (C) is also made from the precipitation, as follows: Cf—Subtropical or Oceanic (humid climate, occurrence of precipitation in all months of the year and absence of defined dry season), Cs—Mediterranean (Winter rain), and Cw—Monsoon-influenced humid subtropical climate (Summer rain). The third letter is used to distinguish climates with different air temperature variations and in the hot temperate climate: (a) a hot summer (average air temperature in the hottest month > 22°C) and (b) temperate summer (average air temperature in the hottest month < 22°C and average air temperature in the hottest four months > 10°C).

Agriculture in Brazil has been expanding in the last decades and is currently the sector with greatest contribution to the economy in the country. Irrigated agriculture plays an important role in this growth. The estimated irrigated area in 2014 was 6.11 million hectares, representing 21% of the national potential (29.6 million hectares), increasing at rates higher than the growth of the total planted area [32]. According to the National Water Agency, in the year 2015, in Brazil, 1,210 m^3^ s^-1^ of water were consumed across, considering all sectors, and irrigation accounted for 75% of this total [32].

### Historical weather data

**Fig 1:**
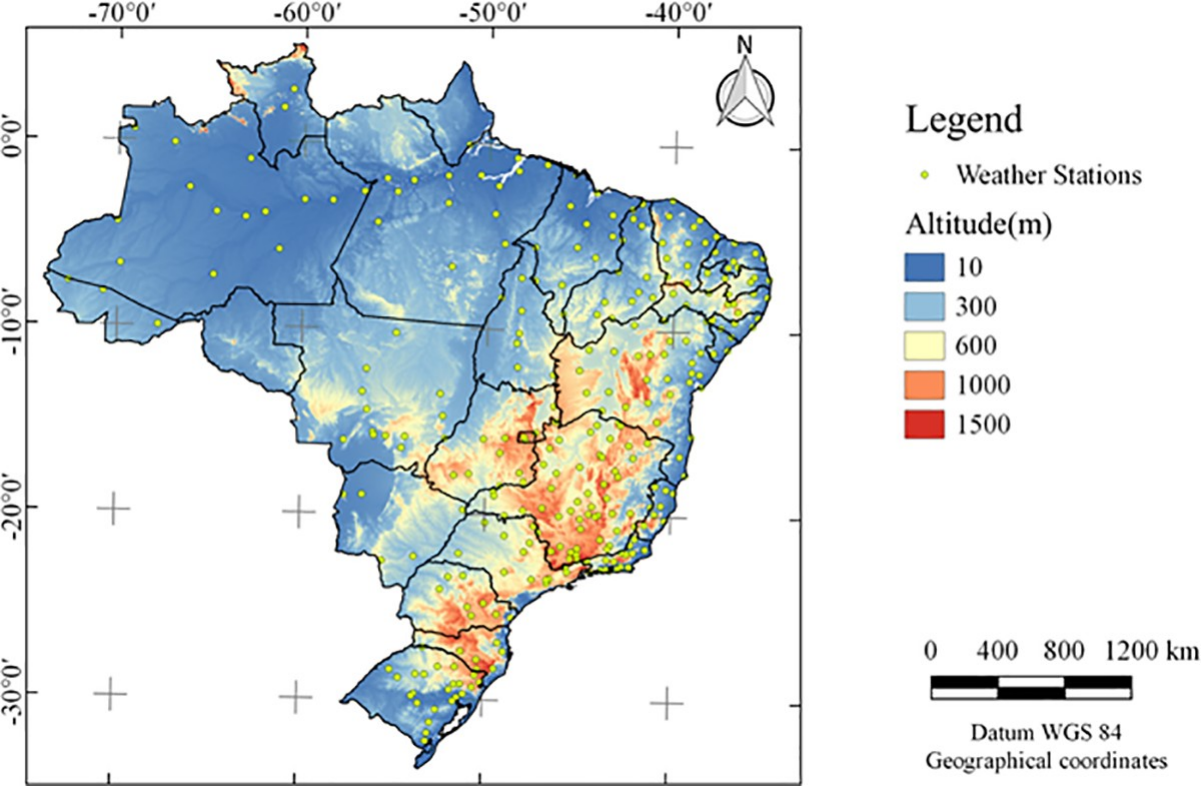
Location of Meteorological Database for Teaching and Research stations of INMET.

The meteorological data needed for the survey were taken from the BDMEP of the INMET. These data are reliable and have been used in several research studies [8, 33, 34]. Data were from January 1, 1961 to December 31, 2016, but not all stations had complete daily data for the entire time series. The data period was longer than those used to perform the checks and possible filling of gaps. The INMET conventional station database had 265 measurement points distributed throughout Brazil (**Fig 1**).

The elevation data used was produced by WorldClim, derived from the SRTM elevation data, with a spatial resolution of 30 seconds, downloaded from https://www.worldclim.org/data/worldclim21.html. The shapefile points were prepared using the geographic coordinates of the Meteorological Stations obtained at http://www.inmet.gov.br/portal/index.php?r=estacoes/estacoesConvencionais. The regional division of Brazil was download from the Brazilian Institute of Geography and Statistics—IBGE (https://www.ibge.gov.br/geociencias/downloads-geociencias.html).

The geographic location of the stations (**Fig 1**) served as a basis for extracting the PET values from the MOD16 product. This extraction was done using the point sampling tool plugin present in QGIS® software [35].

### Calculation of ETo

After obtaining the data from the BDMEP stations, the ETo was calculated using the standard equation (Eq 1), recommended in the FAO 56 bulletin [9]. This methodology assumes the ET of a hypothetical grass field with a height of 0.12 m, aerodynamic resistance of 70 s m^-1^, and albedo of 0.23.

$$\mathrm{ETo}=\frac{0.408\;\Delta (R_{n}-G)+\gamma \frac{900}{T+273}u_{2}(e_{s}-e_{a})}{\Delta +\gamma (1+0.34\;u_{2})}$$ (1)

where ETo is the reference evapotranspiration, mm d^-1^; R_n_ is the surface radiation balance, MJ m^-2^ d^-1^; G is the soil heat flux, MJ m^-2^ d^-1^; T is the mean air temperature, °C; u_2_ is the wind speed at 2 m height, m s^-1^; e_s_ is the saturation vapor pressure, kPa; e_a_ is the current vapor pressure of the air, kPa; Δ is the slope of saturation vapor pressure curve, kPa °C^-1^; and γ is the psychrometric coefficient, kPa °C^-1^.

Due to the large amount of data needed for the calculation of ETo, the software CLIMA®, developed by Faria et al. [36] at the Agronomic Institute of Paraná (IAPAR) in Brazil, was used. ETo was calculated by the FAO-56 Penman-Monteith method and the gaps were filled using methods already validated and used by several authors [37–39]. Besides the calculation and gap-filling of meteorological data, the software checks for quality and data abnormality, from pre-established values. The daily ETo data of the stations were integrated monthly to be used as a dependent variable in the modeling.

### The MOD16 product

MOD16 product is dataset that include the global evapotranspiration (ET), latent heat flow (LE), potential ET (PET) and potential LE (PLE). The MOD16 product provides regular 1-km^2^ land surface ET datasets for the 109.03 Million km^2^ global vegetated land areas at 8-day, monthly and annual intervals. The algorithm described by Mu et al. [26], improved from Mu et al. [21], uses the Penman-Monteith approach [40], combining remote sensing data with reanalysis of meteorological data to calculate plant and canopy transpiration, as well as soil evaporation. The MODIS input data required for the MOD16 algorithm includes global soil and land cover products (MOD12Q1), leaf area index (LAI), fraction of photosynthetically active radiation (FPAR-MOD15A2), and albedo (MCD43B2) [30].

In this study, monthly data were used. The MOD16 data were available in the sinusoidal projection, so it was necessary to reproject them and define the datum. To facilitate the handling, the data were converted from Hierarchical Data Format (HDF) to Geographic Tagged Image File Format (GeoTIFF). This entire process was carried out using MODIS reprojection tools (MRT) [41, 42]. From the available data of the MOD16 product, PET was the only variable used.

To download, the images were standardized with the orbits of their respective points (tiles). Based on the monthly products, images corresponding to the period from January 1, 2000, to December 31, 2014, were used. To cover the Brazilian territory, the tiles used were h10v08, h10v09, h11v08, h11v09, h11v10, h12v08, h12v09, h12v10, h12v11, h13v08, h13v09, h13v10, h13v11, h13v12, h14v09, h14v10, and h14v11. Thus, 12 monthly images were used during 15 years, with 17 different tiles per month, totaling 3,060 images of the MOD16 product, with downloads made at the link: http://files.ntsg.umt.edu/data/NTSG_Products/MOD16/.

MOD16 PET does not cover all land uses, only those that have vegetation. Therefore, for the other uses the values of the pixels of the images are filled with the following codes: Earth (bare soil and rock), 32767; body of water, 32766; barren or sparse vegetation, 32765; permanent snow and ice, 32764; permanent wetlands, 32763; urban or built, 32762; unlisted, 32761 [43].

### WorldClim dataset

The WorldClim product developed by Fick and Hijmans [44] is a set of global climate layers, with a spatial resolution of about 1 km^2^. The WorldClim dataset were generated for 1970–2000, using data from 9,000 to 60,000 weather stations. WorldClim data is available for download at http://worldclim.org/. The covariables used are shown in Table 1.

**Table 1:** WorldClim covariates used in the study.

Variable	Description
Bio01	Annual mean temperature
Bio02	Mean diurnal range
Bio03	Isothermality
Bio04	Temperature seasonality
Bio05	Max temperature of warmest month
Bio06	Min temperature of coldest month
Bio07	Temperature annual range
Bio08	Mean temperature of wettest quarter
Bio09	Mean temperature of driest quarter
Bio10	Mean temperature of warmest quarter
Bio11	Mean temperature of coldest quarter
Bio12	Annual precipitation
Bio13	Precipitation of wettest month
Bio14	Precipitation of driest month
Bio15	Precipitation seasonality
Bio16	Precipitation of wettest quarter
Bio17	Precipitation of driest quarter
Bio18	Precipitation of warmest quarter
Bio19	Precipitation of coldest quarter

### Regression algorithms and modeling

To perform the modeling of ETo, eight regression algorithms were tested: Multiple Linear Regression—LM [45]; Random Forest—RF [46]; Cubist [47]; Partial Least Squares—PLS [48]; Principal Components Regression—PCR [49]; Adaptive Forward-Backward Greedy—FoBa [50]; Generalized Boosted Regression—GBM [51], and Generalized linear model by likelihood-based boosting—GLMboost [52].

Each algorithm tested has its characteristics, advantages, and disadvantages of use. However, because it is a large number of models tested in the present study (8 models), only those that performed well will be detailed below.

Multiple Linear regression aims to find the linear function that minimizes the sum of the squares of errors (SSE) between the observed and predicted data. An advantage of this method is the easy interpretation of the coefficients that are generated in the model, besides having a low computational cost in comparison to the others [47].

The Cubist model implements a regression tree algorithm, which combines instance- based and model-based techniques to create rule-based multivariate regression models from training data. This model has the characteristics of being based on multiple regression models, so that the final product is the average of all of them. The Cubist model has been widely used in applications for remote sensing data [53].

Random forest is an aggregation of trees dependent on random variables. For example, bagging trees (building trees on random subsets of predictors and bootstrap samples of the training data) defines a random forest. The Random Forest allows to improve the predictive accuracy and to control over-fitting [54].

PLS linearize models that have nonlinear parameters. Therefore, it was performed as an adaptation in the regression methodology of the NIPALS algorithm so that it was able to perform regressions with correlated predictors. This modification was denoted as PLS [47, 55].

PCR is an adaptation of LM, which tries to solve the high correlation of the predictors, performing a principal component analysis (PCA) of them, that is, the predictors are preprocessed via PCA. Use this algorithm is recommended when there are variables that have a high correlation among them or for cases that have more variables than observations [47, 55].

GBM is known as one of the most robust prediction techniques, which has come forward with the idea of modifying a weak predictor to become more efficient. This boosting method uses the creation of a set of weak predictors in sequences; most of the time, these predictors are decision trees [56].

**Fig 2:**
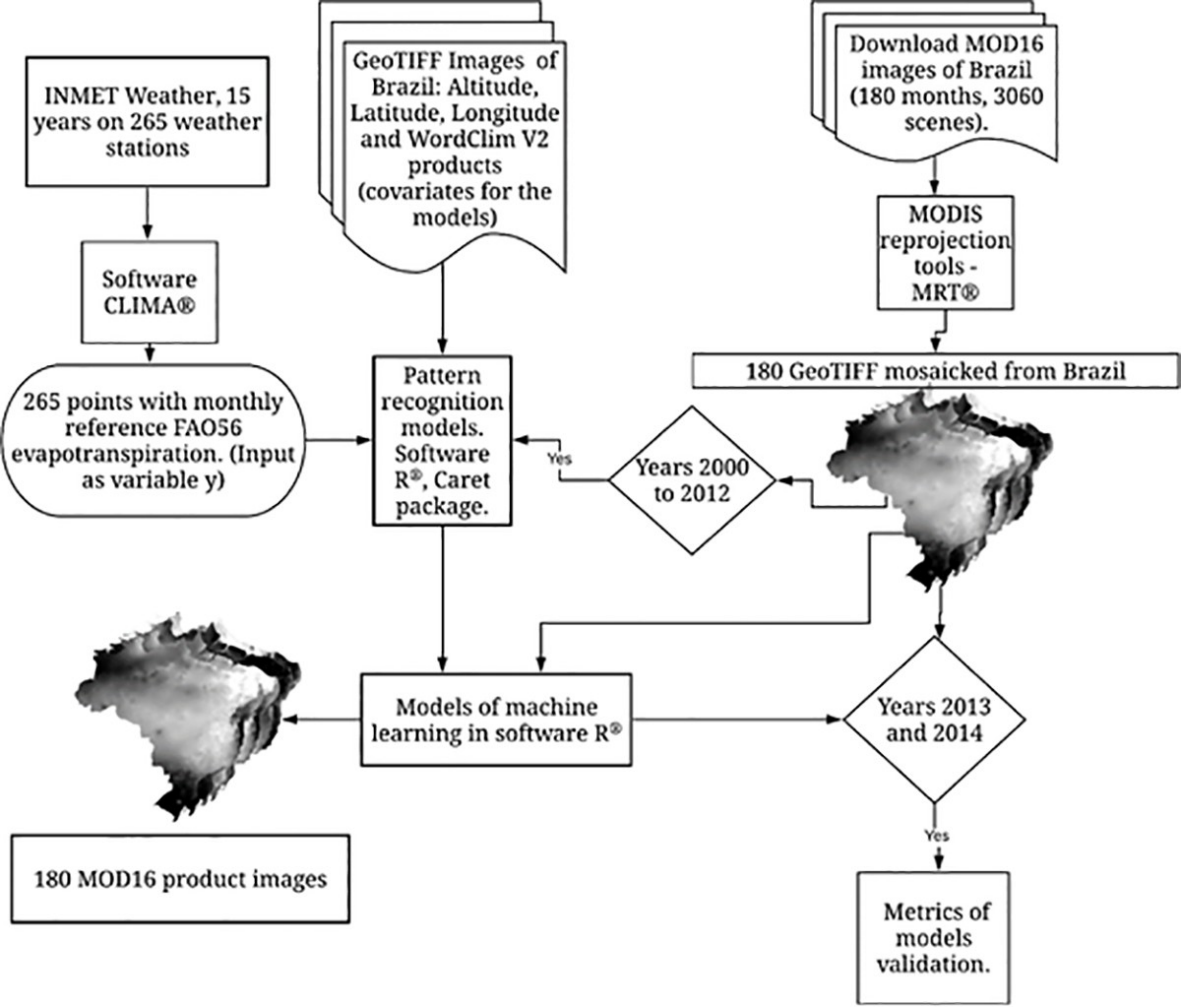
Flowchart of the methodology used to model the ETo. The figure was elaborated with the TIFF files processed in the paper using the free software QGIS [35].

In order to carry out the modeling, it was necessary to calculate the monthly ETo (target variable) of the stations (**Fig 2**), since the MOD16 product is monthly.

Among the bioclimatic variables of WorldClim V2 [44] and MOD16, the covariates with the greatest influence on the models were selected. For this purpose, a function called Recursive Feature Elimination (RFE), present in the labgeo Package [57], was used inside the R software. With this feature it is possible to select the metric that will be used to select the ideal model. By default, possible values are "RMSE" and "Rsquared" for regression and "Accuracy" and "Kappa" for classification. Thus, it was possible to eliminate the highly correlated explanatory variables and rank and select the most important explanatory variables to be used in the modeling. The most important covariates selected in this methodology are presented in order of importance in Table 2.

**Table 2:** Variables selected for training of the models according to the Recursive Feature Elimination.

Variable	Description
MOD16 PET	Potential evapotranspiration (mm per month)
Srad	Solar radiation (kJ m^-2^ d^-1^)
Prec	Precipitation (mm)
Wind	Wind speed (m s^-1^)
Bio02	Mean Diurnal Range (Mean of monthly (max temp-min temp)) (°C)
Bio12	Annual Precipitation (mm)
Bio19	Precipitation of Coldest Quarter (mm)
Bio13	Precipitation of Wettest Month (mm)
Altitude	Altitude WorldClim (m)
Bio07	Temperature Annual Range (°C)
Bio15	Precipitation Seasonality (Coefficient of Variation)

In the present study, we used the caret package [58] on the statistical software R to train all the regression algorithms used. The caret package uses in the training function the grid search by default to fit the parameters of each algorithm in the training. To train the models using this package, first we have to choose the set of parameters of each regression to analyze (tuning parameters). After, we have to specify the type of resampling, which in this study was the k-fold cross-validation.

Wing et al. [58] describe all the training process, cross-validation performed by the models and their respective regression strategies. However, the cross validation supported in the caret was not used to choose the better model to predict the ETo in the present research, we only consider this cross-validation to better tune the parameters. To decide the best model we perform a cross-validation denominated holdout and we repeated this procedure 100 times, where two-year subsets of the data were separated randomly to perform the validation. Thus, the training and validation was carried out randomly 100 times differently, and the mean of the statistical indices was used as the value for the models.

As shown in **Fig 2**, which exemplifies a training performed, 13 years (86.67%) of data were used to perform the training (training set) and two years (13.33%) of data for the validation (validation set) of the models. From the daily ET data from stations, the monthly data were obtained. Spatial and temporal separation was tested, however there was no difference in the fit for the different separation methods. It was decided to use temporal separation with training for 13 years and test with 2 years. Despite the test being carried out with data of 2 years, spatially we had a large area with great variability of climate, altitude, and vegetation, among others.

### Statistical analyses

Statistical evaluations were carried out based on statistical indices with different functions. The coefficient of determination (r^2^) indicates a descriptive measure of the quality of fit obtained, that is, how much the model was able to account for the variability of the observed data. However, it does not take into account the lack of fit, which could be large, especially if the observed and predicted values were non-linearly related. Thus, r^2^ should not be considered alone, but should generally be combined with other metrics.

The root mean square error (RMSE) provides a measure of the mean magnitude of the error through the squared difference between the estimated and observed data. The normalization of the root mean square error (nRMSE) provides a measure of the mean magnitude of the error. Unlike RMSE, normalization allows errors to be observed, regardless of the magnitude of the variable of interest. The mean absolute error (MAE) gives a mean value of the absolute errors. The RMSE gives a greater weight to the large errors, and thus, comparing it with the MAE can indicate the presence of outliers, which is useful when large errors are particularly undesirable [59, 60].

The mean bias error (MBE) can indicate tendencies of underestimation or overestimation. The Nash-Sutcliffe (NSE) efficiency is used to evaluate the predictive power of the model and varies from -∞ to 1, with 1 being the perfect fit between the data estimated by the model and the measured data [61, 62].

Eqs 2 to 7 represent the statistical indices:

$$\mathrm{RMSE}=\sqrt{\frac{\sum_{i=1}^{n}{\left(O_{i}-P_{i}\right)}^{2}}{n}}$$ (2)

$$\mathrm{nRMSE}=\frac{\sqrt{\frac{\sum_{i=1}^{n}{\left(O_{i}-P_{i}\right)}^{2}}{n}}}{\bar{O}}\times 100$$ (3)

$$\mathrm{NSE}=1-\frac{\sum_{i=1}^{n}{\left(O_{i}-P_{i}\right)}^{2}}{\sum_{i=1}^{n}{\left(O_{i}-\bar{O}\right)}^{2}}$$ (4)

$$\mathrm{MAE}=\frac{1}{n}\sum_{i=1}^{n}|P_{i}-O_{i}|$$ (5)

$$\mathrm{MBE}=\frac{1}{n}\sum_{i=1}^{n}\left(P_{i}-O_{i}\right)$$ (6)

$$r^{2}=\frac{{\left(\sum_{i=1}^{n}\left(P_{i}-\bar{P})(O_{i}-\bar{O}\right)\right)}^{2}}{\left(\sum_{i=1}^{n}(P_{i}-\bar{P}{)}^{2})(\sum_{i=1}^{n}(O_{i}-\bar{O}{)}^{2}\right)}$$ (7)

where *P*_*i*_ is the value predicted by the model, mm per month; *O*_*i*_ is the observed value; $\bar{P}$ is the average value predicted by the model; *Ō* is the average observed value; and *n* is the number of samples.

## Results and discussion

**Fig 3:**
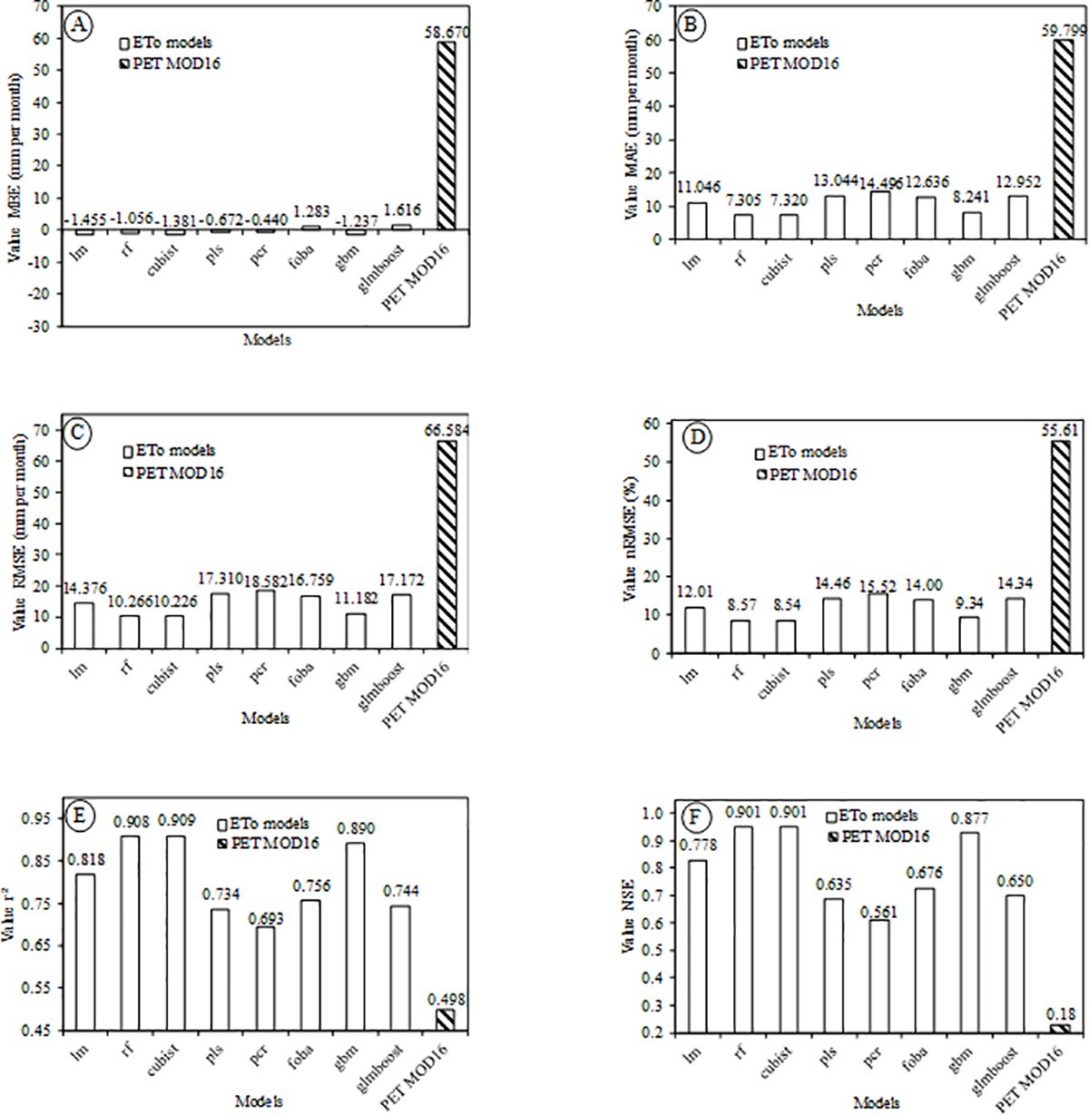
Results of the statistical indices used to evaluate the pre-selected models in comparison to the standard method (PM-FAO), (A) MBE, (B) MAE, (C) RMSE, (D) nRMSE, (E) r^2^ and (F) NSE with out-of-sample data from the period 2012–2013.

**Fig 3** shows the statistical indices of the MOD16 PET and the ETo modeled by different regression algorithms in comparison to the standard ETo method (PM-FAO). The MOD16 PET variable was added to the error graphs just for comparison with the ETo results generated by the different models, since they are different variables. Thus, as expected, the values of the statistical indices for PET of MOD16 product clearly differed from all the modeled ETo methods.

**Fig 3** shows that all the algorithms used in the ETo modeling presented MBE values below 1.62 mm per month. This indicates that the values overestimated and underestimated by the methods were close, but yet, some methods such as the Cubist and linear regression methods tended to underestimate the ETo values. The foba, glmboost, and MOD16 models overestimated ETo.

According to Yao et al. [63], algorithms based on machine learning processes have been widely used to estimate evapotranspiration, but when used uniquely in the estimation of parameters, they still have an uncertainty in their prediction. In this study, the support vector machine (SVM), Bayesian Model Averaging (BMA), and General Regression Neural Networks (GRNNs) were implemented to improve ET results estimated by three process-based ET algorithms: MOD16 (MODIS ET products algorithm), PT-JPL (Priestley-Taylor ET algorithm of Jet Propulsion Laboratory), and SEMI-PM (Semi-empirical Penman ET algorithm). These authors verified that in the ET results analyzed, the SVM was the model that stood out the most against the others for considerably reducing the errors.

After the modeling of ETo, the MAE was drastically reduced, that is, the models of ETo, when compared to the MOD16 PET, obtained results closer to those observed at the meteorological stations (**Fig 3B**). A similar result was obtained when the RMSE (**Fig 3C**) and nRMSE metrics (**Fig 3D**) were applied, indicating an accuracy gain (all algorithms) of the modeled ETo, against the result of the MOD16. The nRMSE values of all models evaluated were less than 15.6% and MOD16 PET was 55.6% (**Fig 3D**).

Evaluating the MOD16 product for irrigated rice crop in Rio Grande do Sul, Brazil, Souza et al. [64] found an RMSE of 15.87 mm (8d^-1^), which is consistent with the value of 66.584 mm per month found in the present study for the PET of MOD16. After the modeling, the RMSE values decreased significantly compared to the MOD16 PET, which made the product of ETo (all algorithms) more accurate and reliable for use throughout the Brazilian territory for purposes related to reference evapotranspiration. Ramoelo et al. [65], in order to validate the MOD16 product from the flux towers in South Africa’s Savanna, concluded that the product is inefficient and its accuracy is not consistent for the period and the places analyzed, which emphasizes the necessity of a fit before applying or creating a more reliable model.

The original MOD16 product had a low coefficient of determination (r^2^) in comparison to the ETo products modeled in the present research (**Fig 3E**). Although this value is small compared to that obtained by the regression models, it is a considerable value for a predictor variable, which demonstrates huge importance of this variable in the prediction models. PCR and PLS had the lowest values of r^2^ among the eight options analyzed. These models should not be recommended to model ETo based on the MOD16 PET product. According to Khosrav et al. [66], PCR and PLS models are recommended only for a set of data that have a high correlation between the independent variables. Maybe, because of these characteristics they had similar responses.

**Fig 3F** shows that the NSE of the MOD16 PET product was much lower than the values of the ETo models created in the present study. NSE values close to zero show that the average ETo value, obtained through data from weather stations, is a forecast equal to that obtained from the model, indicating a poor performance [62].

According to our results, three of the eight algorithms have a high potential of application. These were the Cubist, Random Forest and Linear Regression. The first two because they had the best results of the statistical indices and the third one, because it is simple to replicate, which is in accordance with the parsimony of science.

The equation fitted with the linear regression model is shown below (Eq 8). The wind speed regression coefficient was significant at 1% and the others at 0.1% by the student’s t-test.

$$ETo=‐27.09344^{**}+0.02381^{**}\;MOD16+0.00594^{**}\;Rad+0.02258^{**}\;Prec‐0.50952^{**}\;Wind+0.85620^{*}\;Bio02+0.00957^{**}\;Bio12+0.01134^{**}\;Bio19‐0.11342^{**}\;Bio13‐0.01267^{**}\;Altitude‐0.89244^{**}\;Bio07+0.41986^{**}\;Bio15$$ (8)

r^2^ = 0.7528; p-value<0.0001; *p<0.01 and **p<0.001

The model showed good generality, since we must consider that our area is very large and presents changes in climate, land use, and altitude, among others. These results answer the suspicions of Jovanovic et al. [27], who believed that MOD16 could be used to estimate ETo in regions with different weather conditions. But we have to agree that due to the relatively coarse resolution of ~1 km^2^ pixels may have implications for applications in restricted areas, especially in heterogeneous vegetation, land use/cover and landscape.

We can emphasize the linear regression in the present study, due to the easy explanation of the results generated and the performance that it reached for ETo modeling in the present study. Westerhoff [30] worked with the precision of the MOD16 product, performing the PET correction with linear regression, and its results were very similar to those obtained in this study.

The RF and Cubist models led to better results for the five most significant indices to evaluate the accuracy of a model. Other research corroborates our results. Noi et al. [67] applied the RF and Cubist models to estimate the daily air surface temperature in northwest Vietnam. For this, the linear/linear multiple regression (LM) algorithms are frequently applied. They found that the Cubist and RF results were similar or far superior to those of the LM and showed better results in all the 15 combinations made. Other authors had already proposed hybrid algorithms between the RF and the Cubist, in order to improve the results that were obtained by the models [68].

Cubist is a powerful tool for generating rule-based models that balance the need for accurate prediction against the requirements of intelligibility. Cubist models generally give better results than those produced by simple techniques such as multivariate linear regression, while also being easier to understand than neural networks.

The cubist algorithm stands out from the RF in time of processing, requiring a shorter time to model the ETo variable. Frondana [69] evaluated the processing time of 16 regression algorithms and 59 datasets sizes. The author found that the larger is the dataset, the better is the performance of the Cubist model in relation to the RF. Thus, the next results refer to the Cubist model, as it showed the best result in modeling the ETo.

**Fig 4:**
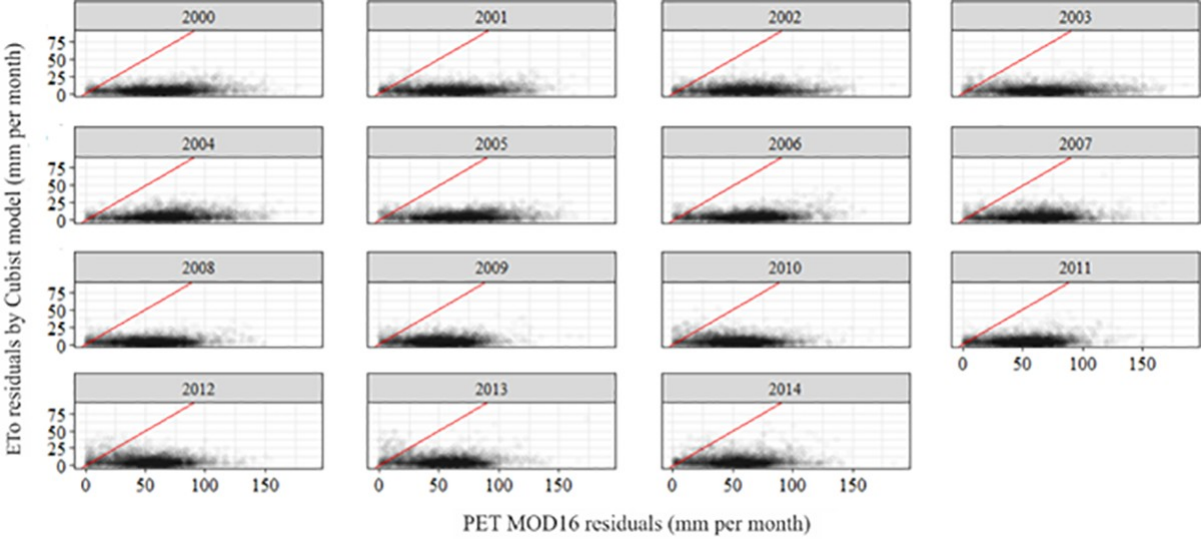
Yearly residual of MOD16 PET compared to the yearly residual of ETo modeled by Cubist algorithm.

**Fig 4** shows the comparison of the residues of the MOD16 PET and ETo products modeled by the Cubist algorithm in all the years analyzed. The residual represents the difference of the product modeled by the Cubist algorithm and the MOD16 PET product, in comparison to the estimated ETo (PM-FAO56) from meteorological station data.

Residuals from MOD16 PET (X-axis) have a high dispersion when compared to the dispersion of the product residual modeled by the Cubist algorithm (Y-axis). The red line is the 1:1 line and shows that the accuracy of the ETo modeled by the Cubist algorithm is much higher than those of the MOD16 PET over the years and the images analyzed.

Table 3 shows the difference between the residuals of the ETo Cubist product and the MOD16 PET product compared with meteorological station.

**Table 3:** Residuals of MOD16 PET and the values predicted by Cubist model in relation to the observed data (weather stations).

Quartiles	MOD16 (mm per month)	Cubist Model (mm per month)
Minimal residual	0.00 (0.00%)	0.00 (0.00%)
First quartile	39.30 (32.83%)	2.16 (1.80%)
Median	58.50 (48.86%)	4.77 (3.98%)
Mean Residual	58.62 (48.96%)	6.36 (5.31%)
Third quartile	76.30 (63.73%)	8.80 (7.35%)

**Fig 5:**
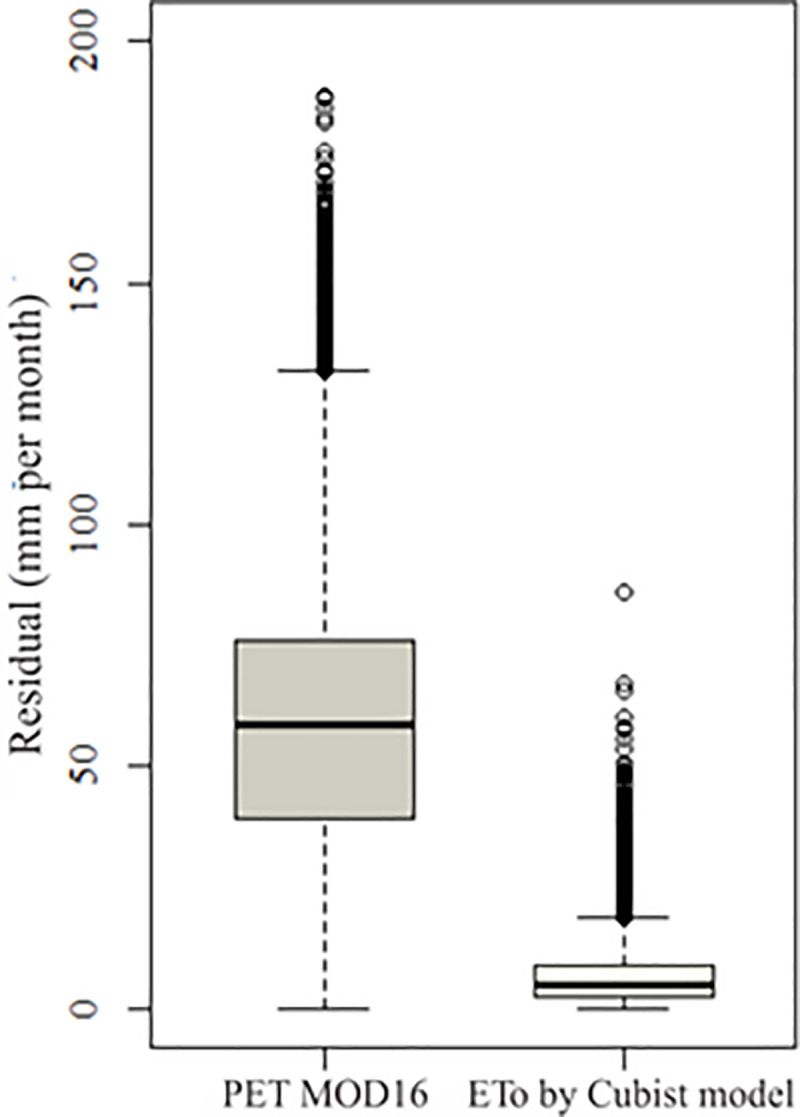
Box plot of the residuals observed in the MOD16 PET product and the ETo modeled by Cubist.

The disparity between the ETo Cubist data versus MOD16 PET data is observed in Table 3. The values can also be observed in the box plot of **Fig 5**, which shows the distribution of all errors in the MOD16 PET and the ETo Cubist product. As expected, the error of prediction of ETo (Cubist) is smaller when compared to the error of the MOD16 product (**Fig 5**), demonstrating the need to correct the MOD16 product before use in Brazil, for ETo applications.

**Fig 6:**
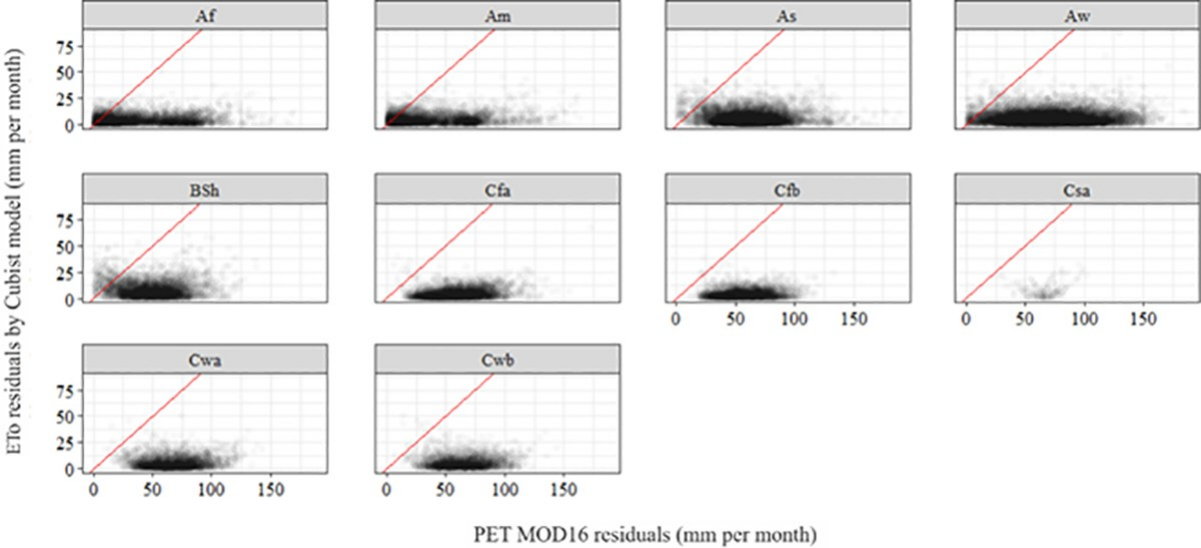
Residual of MOD16 PET x residual of ETo (Cubist model) according to Köppen’s classification. The figure was elaborated using the R software, according to the Köppen climate classification for each season.

**Fig 6** shows the deviations of MOD16 PET and ETo Cubist, according to Köppen’s classification in Brazil. For this analysis, ten climatic classifications were considered, according to Alvares et al. [31]. This analysis was critical to assess the trends of residual distribution, to see if they were being influenced by some aspect of the landscape that was prevalent in each climate. There was a strong tendency to reduce residues for the Cubist model in the C classifications (temperate or hot temperate), where the temperate region begins.

**Fig 7:**
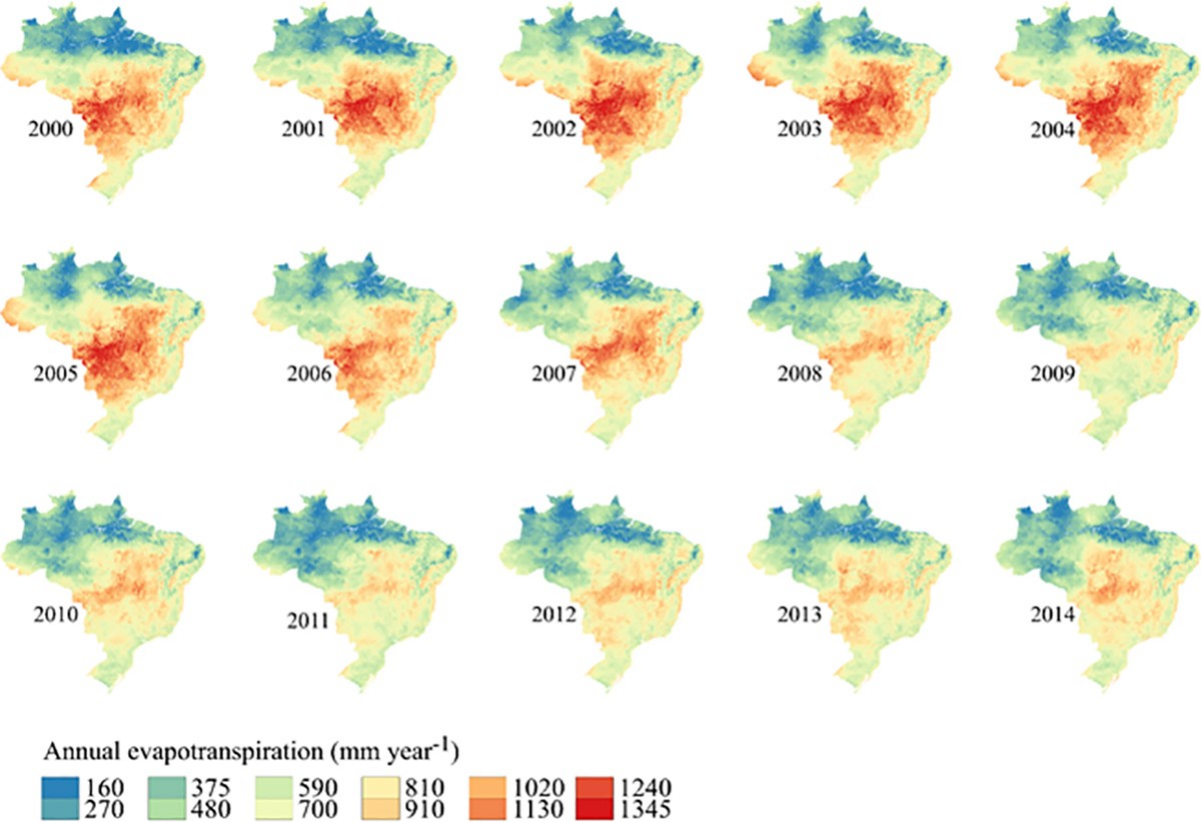
Difference between the annual values of MOD16 ETP and ETo obtained by the Cubist model over the 15 years. The figure was elaborated with the TIFF files processed in the paper, and using the free software QGIS [35].

The differences between MOD16 PET product and ETo cubist are explicit when analyzed in **Fig 7**, which shows the differences in an annual base during the 15 years. The Cubist is a ‘tree’ model and is considered to be a “black box”, where regressions are made at the end nodes. The use of this model is justified in the search for more precise values, which have better fit in their use. The ETo modeled by Cubist improved the accuracy, reducing the error by 4.15 mm per month when compared to linear regression, demonstrating a significantly higher quality compared to other models already used to improve MOD16 performance [30] and algorithms used to model ETo.

It can also be seen in Fig 7 that the difference between MOD16 ETP and ETo Cubist values varied between 160 and 1345 mm per year. The difference was greater in the central part of Brazil, where Aw (Tropical savanna climate with dry-winter characteristics) predominates according to the Köppen classification.

It is also notice that the biggest differences between MOD16 ETP and ETo Cubist (**Fig 7**) coincided with areas that had the highest altitudes (**Fig 1**). However, we do not find support in the literature to explain such behavior.

The results found in the present research are highly relevant, since the use of the MOD16 product to estimate ETo allows the consideration of the surface dynamics and is the guarantee of a more accurate estimate of this variable for regions with no meteorological stations.

## Data sharing and distribution

The monthly ETo data set was stored online in a free repository under the CC BY 4.0 license at https://www.zenodo.org/record/3934663 (Dias et al., 2020). It was named as Monthly reference evapotranspiration for Brazil. The spatial resolution of the data is between 30 seconds (~ 1 km2) and temporal resolution of 1 month. The data set grid is in GeoTIFF format, and corresponds perfectly to WorldClim. It uses the WGS84 coordinate reference system (EPSG: 4326).

## Conclusions

Penman-Monteith is the standard method to estimate ETo and there is a great demand for this information all over the Brazilian territory, in sites often distant from weather stations. Thus, free products of remote sensing should be used for this purpose.

The potential evapotranspiration made available by the MOD16 product, in its original form, has values higher than those of ETo for different periods and climatic regions of Brazil. However, the MOD16 product together with the WorldClim covariates can be used to estimate ETo with the aid of machine learning models.

Among the eight models tested and validated, Cubist and Random Forest were the models that obtained the best results in general, and therefore, are the most suitable models for representing the ETo in space and time for the Brazilian territory. However, linear regression is also recommended, since the results found for this simple model were also useful, and the equation was shown in the present article.

The ETo product was created and evaluated in a monthly temporal resolution and spatial resolution of 30 seconds (~ 1 km^2^) in Brazil. The ETo created in this study enables the execution of many other research studies, related to hydrological modeling and water use management, since it showed a strong reliability in comparison with the ETo estimated from the weather stations in all the national territory.

The proposed method improves the ability to use products derived from satellite data with global coverage, in compliance with the PM-FAO standard used locally.

## Supporting information

S1 File Classification of the climate according to Köppen, by Alvares et al. [31]. (TIF)

S2 File Monthly average of ETo for 15 years of the MOD16 product and ETo by the Cubist model. With these maps it was possible to construct the differences between MOD16 PET and ETo Cubist shown in Fig 7.
(TIF)

S3 File Average, standard deviation and variation coefficient of annual evapotranspiration values for ETo cubist and MOD16 PET. (TIF)

S4 File Average, standard deviation and coefficient of variation of the monthly evapotranspiration values for the cubist ETo. (TIF)

S5 File Average, standard deviation and coefficient of variation of the monthly evapotranspiration values for MOD16 PET. (TIF)
