# Supplementary figures and images for: Reference evapotranspiration of Brazil modeled with machine learning techniques and remote sensing
Source: PLoS One. 2021 Feb 9;16(2):e0245834. doi: 10.1371/journal.pone.0245834 (PMC7872264; doi:10.1371/journal.pone.0245834)

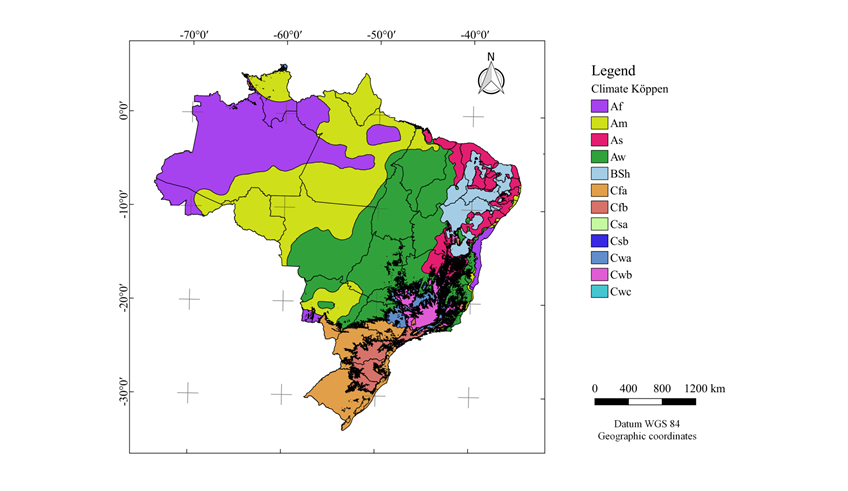

Supplement: S1 File — (TIF) [file pone.0245834.s001.tif]

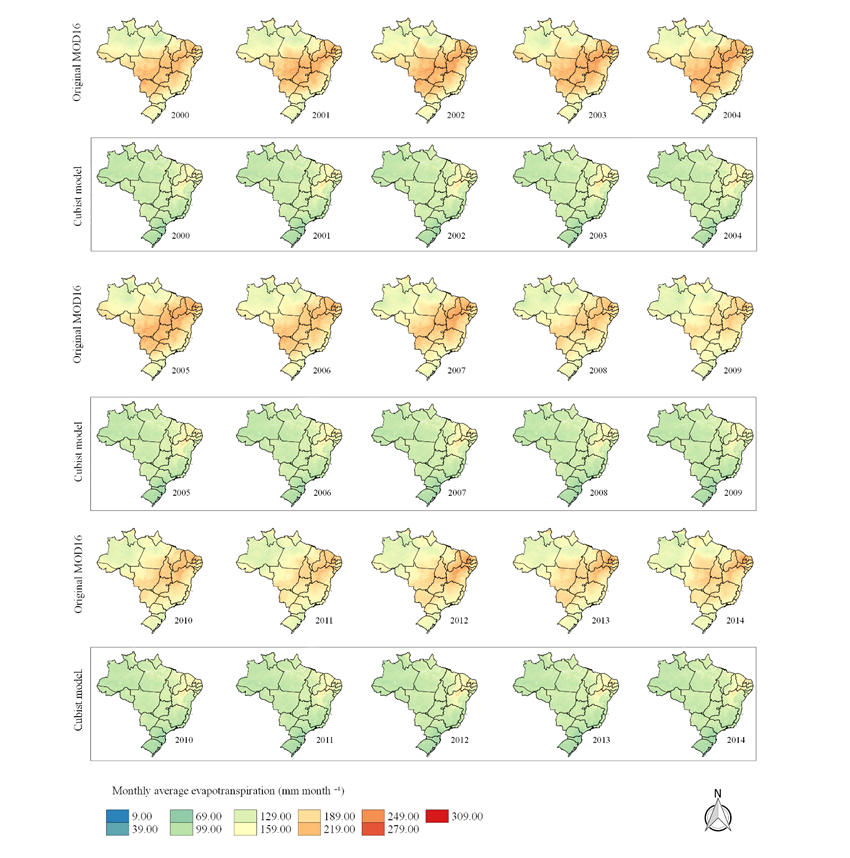

Supplement: S2 File — With these maps it was possible to construct the differences between MOD16 PET and ETo Cubist shown in Fig 7. (TIF) [file pone.0245834.s002.tif]

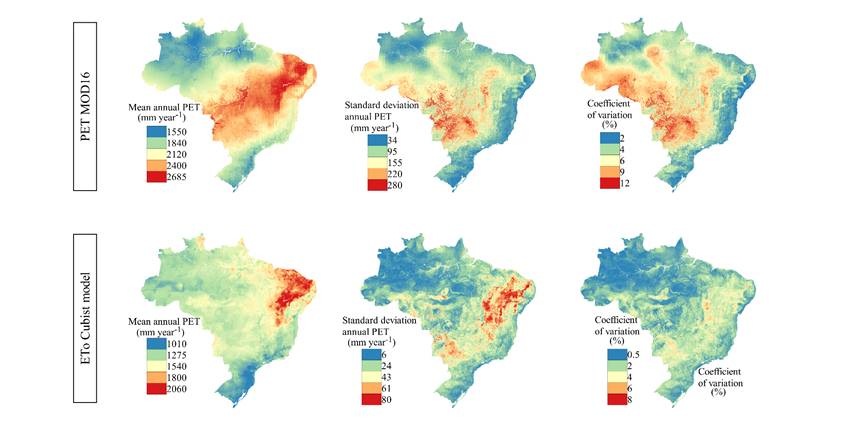

Supplement: S3 File — (TIF) [file pone.0245834.s003.tif]

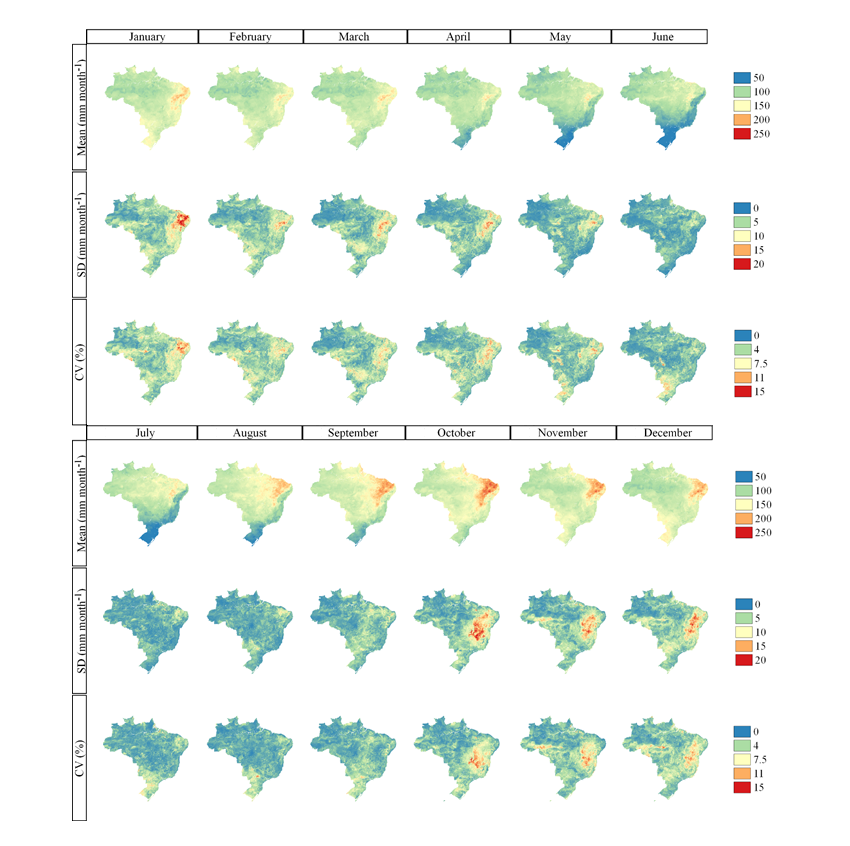

Supplement: S4 File — (TIF) [file pone.0245834.s004.tif]

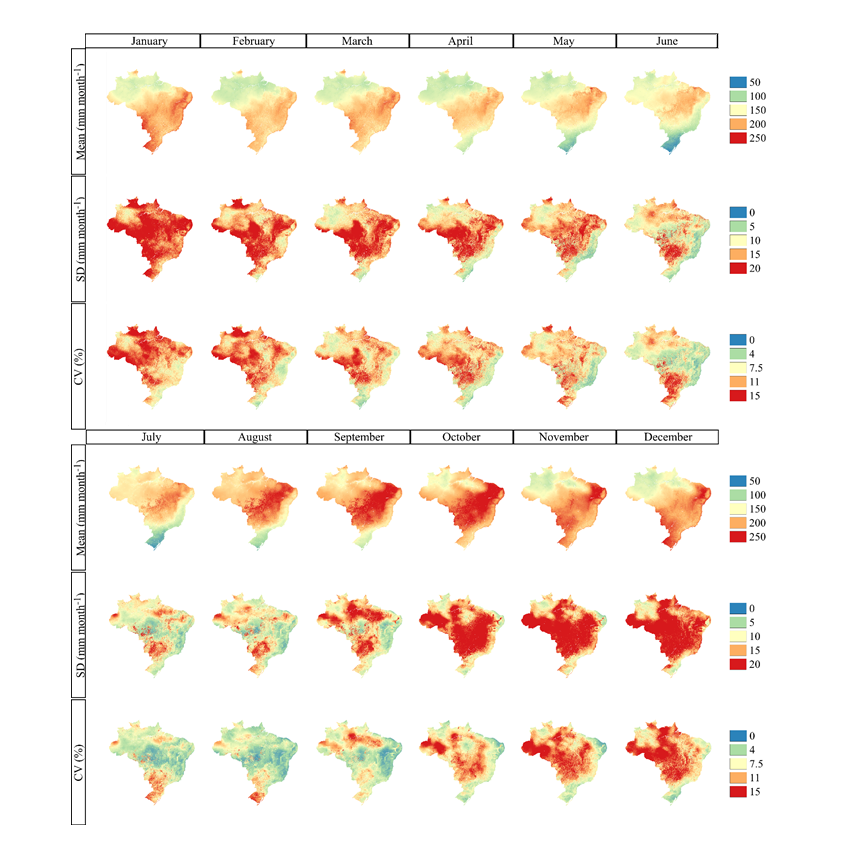

Supplement: S5 File — (TIF) [file pone.0245834.s005.tif]
